# Theoretical Insight Into the Ultralong Room-Temperature Phosphorescence of Nonplanar Aromatic Hydrocarbon

**DOI:** 10.3389/fchem.2021.740018

**Published:** 2021-09-06

**Authors:** Ke Qin, Wenqi Gong, Jia Gao, Deping Hu, Huifang Shi, Wei Yao, Zhongfu An, Huili Ma

**Affiliations:** ^1^ Key Laboratory of Flexible Electronics (KLOFE) & Institute of Advanced Materials (IAM), Nanjing Tech University (Nanjing Tech), Nanjing, China; ^2^ School of Environment, South China Normal University, Guangzhou, China; ^3^ Hefei National Laboratory for Physical Science at the Microscale, University of Science and Technology of China, Hefei, China

**Keywords:** ultralong phosphorescence, organic phosphorescence, nonplanar aromatic hydrocarbon, spin-orbit coupling, vibronic coupling

## Abstract

Purely aromatic hydrocarbon materials with ultralong room-temperature phosphorescence (RTP) were reported recently, but which is universally recognized as unobservable. To reveal the inherent luminescent mechanism, two compounds, i.e., PT with a faint RTP and HD with strong RTP featured by nonplanar geometry, were chosen as a prototype to study their excited-state electronic structures by using quantum mechanics/molecular mechanics (QM/MM) model. It is demonstrated that the nonplanar ethylene brides can offer σ-electron to strengthen spin-orbit coupling (SOC) between singlet and triplet excited states, which can not only promote intersystem crossing (ISC) of S_1_→T_n_ to increase the population of triplet excitons, but also accelerate the radiative decay rate of T_1_→S_0_, and thus improving RTP. Impressively, the nonradiative decay rate only has a small increase, owing to the synergistic effect between the increase of SOC and the reduction of reorganization energy of T_1_→S_0_ caused by the restricted torsional motions of aromatic rings. Therefore, a bright and long-lived RTP was obtained in aromatic hydrocarbon materials with twisted structure. This work provided a new insight into the ultralong RTP in pure organic materials.

## Introduction

Ultralong room-temperature phosphorescence (RTP) in purely organic materials has been gaining more attention in encryption (An et al., 2015; Ma et al., 2021), display (Wang et al., 2019; Tan et al., 2021), bioimaging (Wang et al., 2020; Wang et al., 2021a) and so on (Yu et al., 2017; He et al., 2019; Zhao et al., 2020). Phosphorescence generally refers to the spin-forbidden radiative transition from triplet to singlet states. RTP is common in coordination complexes, which have a lifetime of *μ*s-scale, owing to the increased radiative transition caused by transition metal (eg, Ir, Pt, *etc.*). (Yam et al., 2015). In contrast, pure organic compounds, in principle, have an ultralong phosphorescence lifetime of second-scale, however, their RTP phenomenon is almost unobservable due to the weak spin-orbit coupling (SOC) effect. (TurroRamamurthy and Scaiano, 2010). Namely, the ultralong RTP in aromatic hydrocarbon materials is extremely rare, (Clapp, 1939; Bilen et al., 1978), because of the forbidden intersystem crossing (ISC) process between singlet and triplet excited states. To overcome this issue, the heavy atoms (eg., Br and I) (Cai et al., 2018; Wang et al., 2021b) and carbonyl groups (Zhao et al., 2016; Jia et al., 2020) were incorporated into organic molecules to promote ISC process for achieving ultralong RTP, in combination with the suppression of the nonradiative quenching through rigid environment, (Wu et al., 2020; Zheng et al., 2020; Zhou et al., 2020; Chen et al., 2021; Xie et al., 2021), such as crystal engineering and host-guest system. Very recently, Bechtold *et al.* reported that the nonplanar aromatic hydrocarbon, named as 5,6,11,12,17,18-hexahydrobenzo [2,1-p]chrysene (HD), (Salla et al., 2019), can show an ultralong RTP, which was attributed to the pronounced SOC induced by non-planar configuration. However, the enhanced SOC also reduces the RTP lifetime. Therefore, it is urgent to probe the origin of the ultralong RTP in the nonplanar aromatic hydrocarbons.

The 5′-phenyl-1,1’:3′,1″-terphenyl (PT) has a faint RTP in crystal, while HD shows an efficient RTP with a lifetime of 380 m by introducing saturated ethylene bridges between the central benzene and outer benzene rings of PT. (Li et al., 2015; Salla et al., 2019). Therefore, PT and HD molecules are good prototype to expound the dependence of ultralong RTP on the nonplanar conformation. We thus take these two molecules as example to quantitatively evaluate the nature of the molecular excited states by combing quantum and molecular mechanics (QM/MM) approach and time-dependent density functional theory (TDDFT) coupled with the thermal vibration correlation function (TVCF) formalism and unravel the origin of the ultralong RTP in nonplanar aromatic hydrocarbons, especially the relationship between RTP and the nonplanar conformation.

## Materials and Methods

**FIGURE 1:**
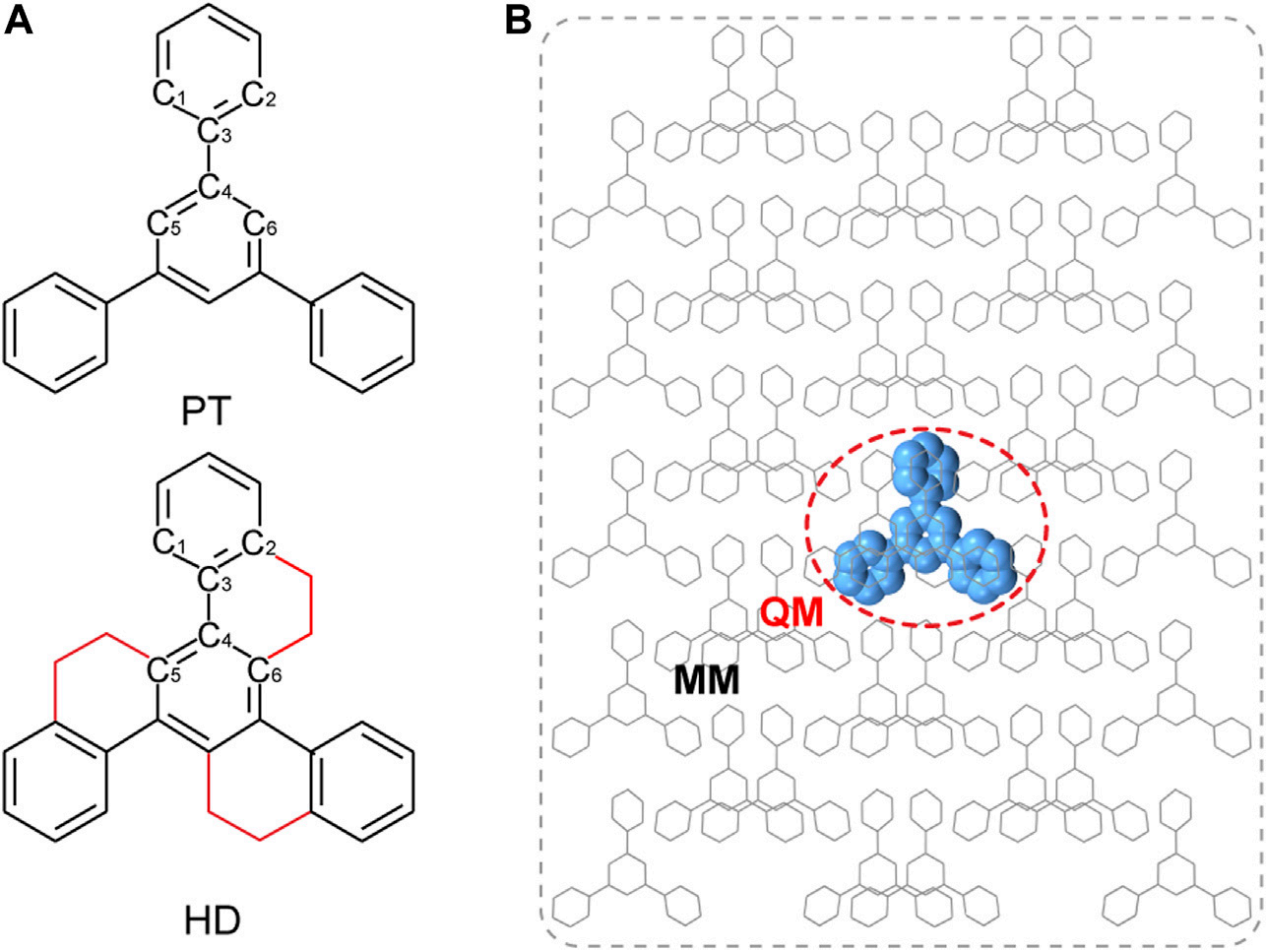
**(A)** Chemical structure of PT and HD; **(B)** Setup of QM/MM models by taking PT as an example.

The QM/MM models were built based on the crystal structures from X-ray single-crystal diffraction as shown in Figure 1B. The central one molecule was chosen as active QM part, while the remaining molecules were defined as rigid MM part, which was performed by using ChemShell 3.7 (Sherwood et al., 2003) packages interfacing ORCA (Neese, 2018) for QM and DL_POLY (Smith and Forester, 1996) with the GAFF (Wang et al., 2004) for the MM part. The geometry optimization and harmonic vibrational frequency of the ground state (S_0_) and the lowest triplet (T_1_) excited states were calculated at (TD) B3LYP/def2-SVP level. Based on the T_1_-geometry, TD-B3LYP/def2-SVP method was used to calculate their electronic structure nature, including excitation energies and natural transition orbitals (NTOs) of the low-lying excited states. All the above calculations were implemented by Gaussian 09 software. (Frisch et al., 2009). At the same level, the SOC matrix elements (ξ) were evaluated by using Beijing Density Function package (BDF) (Liu et al., 1997; Liu et al., 2003; Hirao and Ishikawa, 2004; Li et al., 2013).

The phosphorescence lifetime *τ*_p_ = 1/(*k*_p_ + *k*_nr_) is determined by radiative decay rate *k*_p_ and nonradiative decay rate *k*_nr_. The former can be estimated by Einstein spontaneous emission relationship $k_{p}=\frac{fE^{2}}{1.499\text{ s}\cdot {\text{cm}}^{-2}}$, where ƒ is the oscillator strength, *E* is the vertical excitation energy in wavenumber. While the latter is evaluated by the TVCF rate theory with the Lorentz broadening width of 100 cm^−1^ implemented in MOMAP package. (Niu et al., 2018). It is noted that Duschinsky rotation effect is not considered.

## Results and Discussions

The light-emitting behavior of organic phosphors is governed by the molecular excited-state process. We thus explored the excited-state electronic structure, including excitation energy, NTOs and SOC matrix elements, and excited state decay rates of T_1_→S_0_ to account for the origin the ultralong RTP in nonplanar aromatic hydrocarbons.

### Nature of The Low-Lying Excited States

**FIGURE 2:**
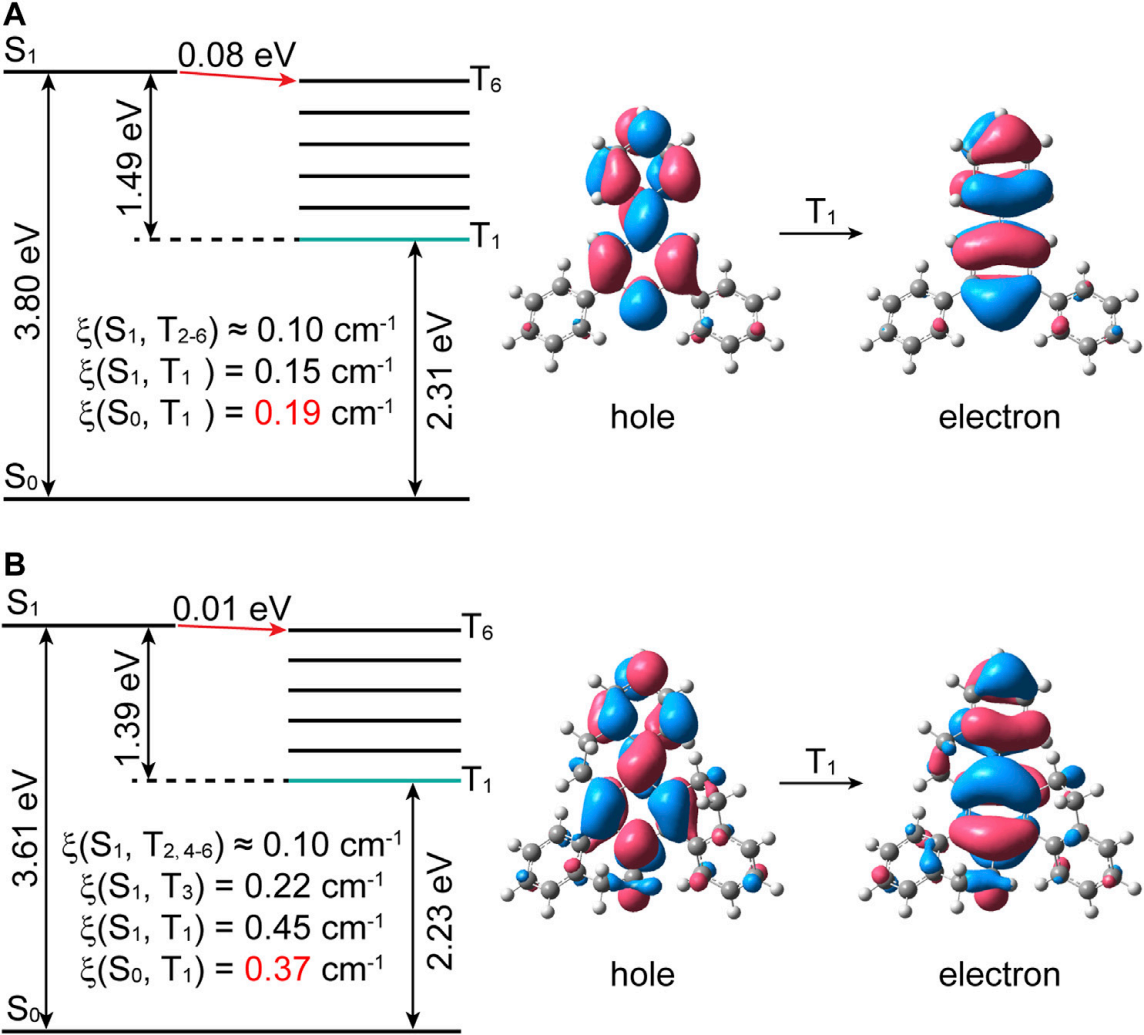
Calculated energy diagram and SOC matrix elements (ξ) of the low-lying excited states, and NTOs of T_1_ states for **(A)** PT and **(B)** HD.

As seen from Figure 2, the energy gap of S_1_→T_6_ is decreased from 0.08 eV in PT to 0.01eV in HD, along with the similar SOC values ξ(S_1_, T_6_). While the ξ(S_1_, T_n_) (n = 1–3) are about twice in HD than in PT molecules, which can be ascribed to the introduction of σ→π^*^ transition (>4.0%) caused by the twisted ethylene bridge in HD (Schmidt et al., 2007), see Supplementary Figure S1 and Supplementary Table S3. Thus, it rationally speculated that the ISC process of S_1_→T_n_ should be largely promoted, which is responsible for the bright RTP in HD. On the other hand, the ξ(T_1_, S_0_) shows an increasing tendency from 0.19 cm^−1^ in PT to 0.37 cm^−1^ in HD, which can be attributed to the increased proportion of σ→π^*^ transition for T_1_ state from 0.0% in PT to 5.47% in HD caused by the twisted ethylene bridge (see Figure 2 and Supplementary Tables S1, S2), and such change is beneficial to the increase of SOC according to El-Sayed’s rule (El‐Sayed, 1963; El-Sayed, 1968). Such enlarged SOC of T_1_→S_0_ in HD not only increases the radiative decay rate *k*_p_, but also largely accelerates the nonradiative decay rate *k*_nr_, making the shorten of the RTP lifetime (Ma et al., 2019). In addition, the excitation energy of T_1_ state is decreased from 2.31 eV in PT to 2.23 eV in HD, where the excitation energy of HD agrees well with the RTP spectra (2.33 eV) in experiment, indicating the promotion of the *k*_nr_. Namely, both the increase of the SOC value and decrease of the energy gap of T_1_→S_0_ are favorable for the acceleration of the nonradiative decay process, and this change is against the ultralong phosphorescence lifetime. Therefore, it is necessary to further expound the effect of vibronic coupling on the ultralong RTP of nonplanar HD compound.

### Excited State Decay Rates of T_1_→S_0_

It is well-known that the phosphorescence lifetime is given by the formula *τ*_p_ = 1/(*k*_p_ + *k*_nr_), where the *k*_p_ can be evaluated by Einstein spontaneous emission relationship, and the *k*_nr_ was calculated by TVCF rate theory using MOMAP program (Niu et al., 2018). Table 1 and Supplementary Figure S2 show that, going from PT to HD, the *k*_nr_ is slightly increased from 4.32 to 9.49 s^−1^, and it dominates the phosphorescence lifetime, because of the slower *k*_p_ with a small enlargement from 3.00 × 10^–2^ to 6.40 × 10^–2^ s^−1^. Consequently, the calculated phosphorescence lifetime is reduced from 0.23 s in PT to 0.11 s in HD. Impressively, the RTP lifetime of HD in experiment (0.38 s) are reproduced by the calculated value of 0.11 m. These results demonstrated that the nonplanar ethylene bridges have a little impact on the ultralong RTP lifetime of HD.

**TABLE 1:** Calculated radiative *k*_p_ and nonradiative decay rates *k*_nr_ of T_1_→S_0_, as well as the RTP lifetime *τ*_p_ = 1/(*k*_p_ + *k*_nr_) for PT and HD. The experimental value was also given as a comparison.


T = 300 K	*k*_p_ (s^−1^)	*k*_nr_ (s^−1^)	*τ*_p_ (s)	
			Cal	Exp
PT	3.00 × 10^–2^	4.32	0.23	—
HD	6.40 × 10^–2^	9.49	0.11	0.38

**FIGURE 3:**
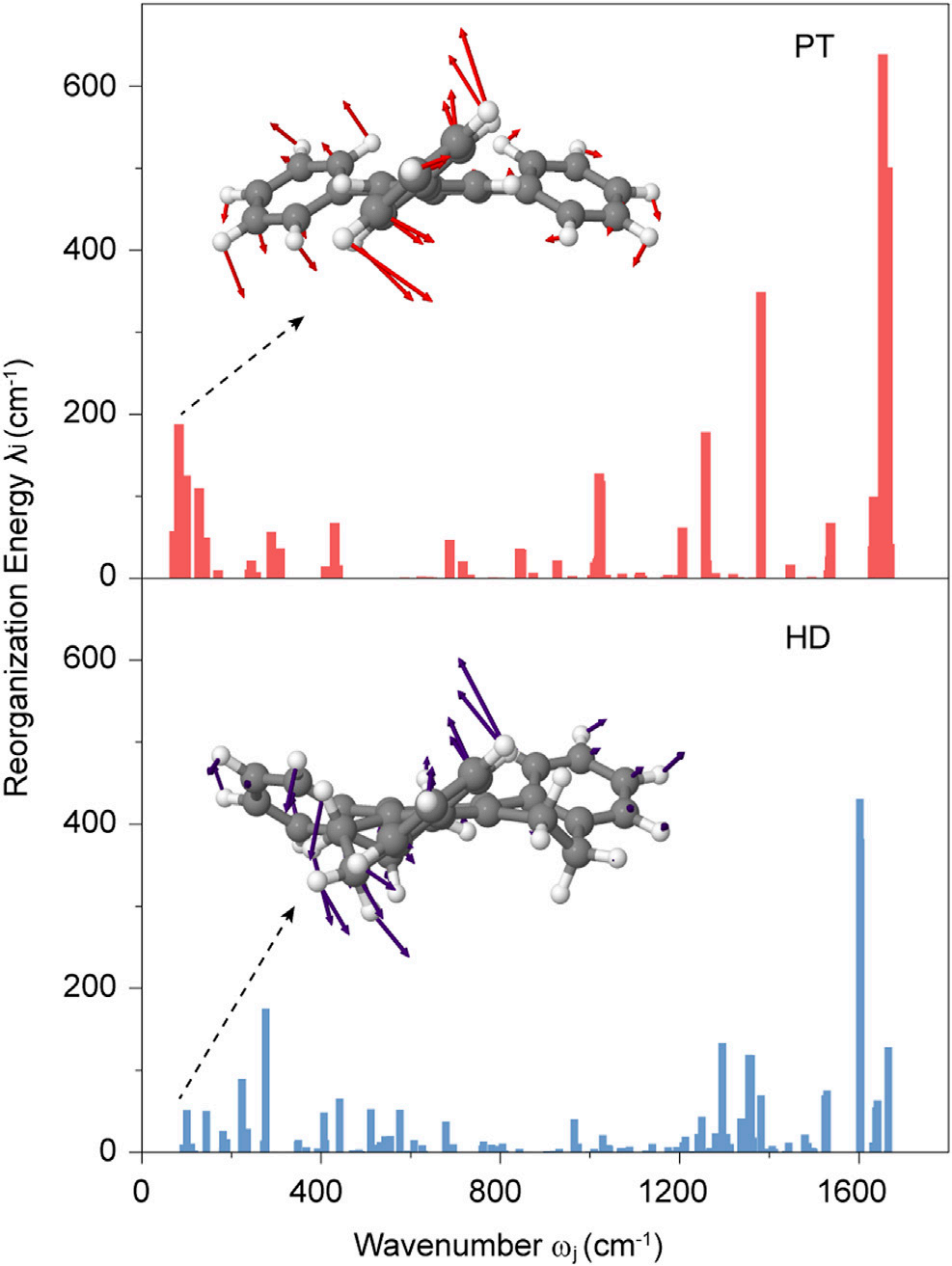
Calculated reorganization energy λ_j_ of T_1_ → S_0_ for PT and HD.

To better understand such a small change, we then focus on the nonradiative decay rate *k*_nr_, which was not only connected with SOC and energy gap *E* of T_1_→S_0_, but also was governed by the electron-vibration coupling characterized by the reorganization energy *λ*. (Marian, 2012). As shown in Figure 3, the total reorganization energy has a large decrease, from 3,624.29 cm^−1^ in PT to 3,096.66 cm^−1^ in HD. Such a change is primarily originated from the reduced *λ* in low-frequency regions (ω < 300 cm^−1^) from 827.37 cm^−1^ in PT to 307.30 cm^−1^ in HD, which are mainly associated with the torsional motions of benzene rings. Additionally, the *λ* in high-frequency regions (∼1,600 cm^−1^) relating to the C=C stretching vibration also provide a significant contribution, decreasing from 1,388.87 cm^−1^ in PT to 1,076.33 cm^−1^ in HD (see Supplementary Figure S3). These results demonstrated that the nonplanar ethylene bridges in HD can reduce the *λ*, resulting in the decrease of *k*_nr_. It is worthwhile note that this change is conflict to the acceleration of the *k*_nr_ due to the increased SOC and reduced energy gap of T_1_→S_0_ caused by the nonplanar ethylene bridges (see Figure 2). Therefore, the *k*_nr_ has only a slight increase from PT to HD, owing to the balance of the change in SOC, *E* and *λ* caused by the nonplanar ethylene bridges.

**FIGURE 4:**
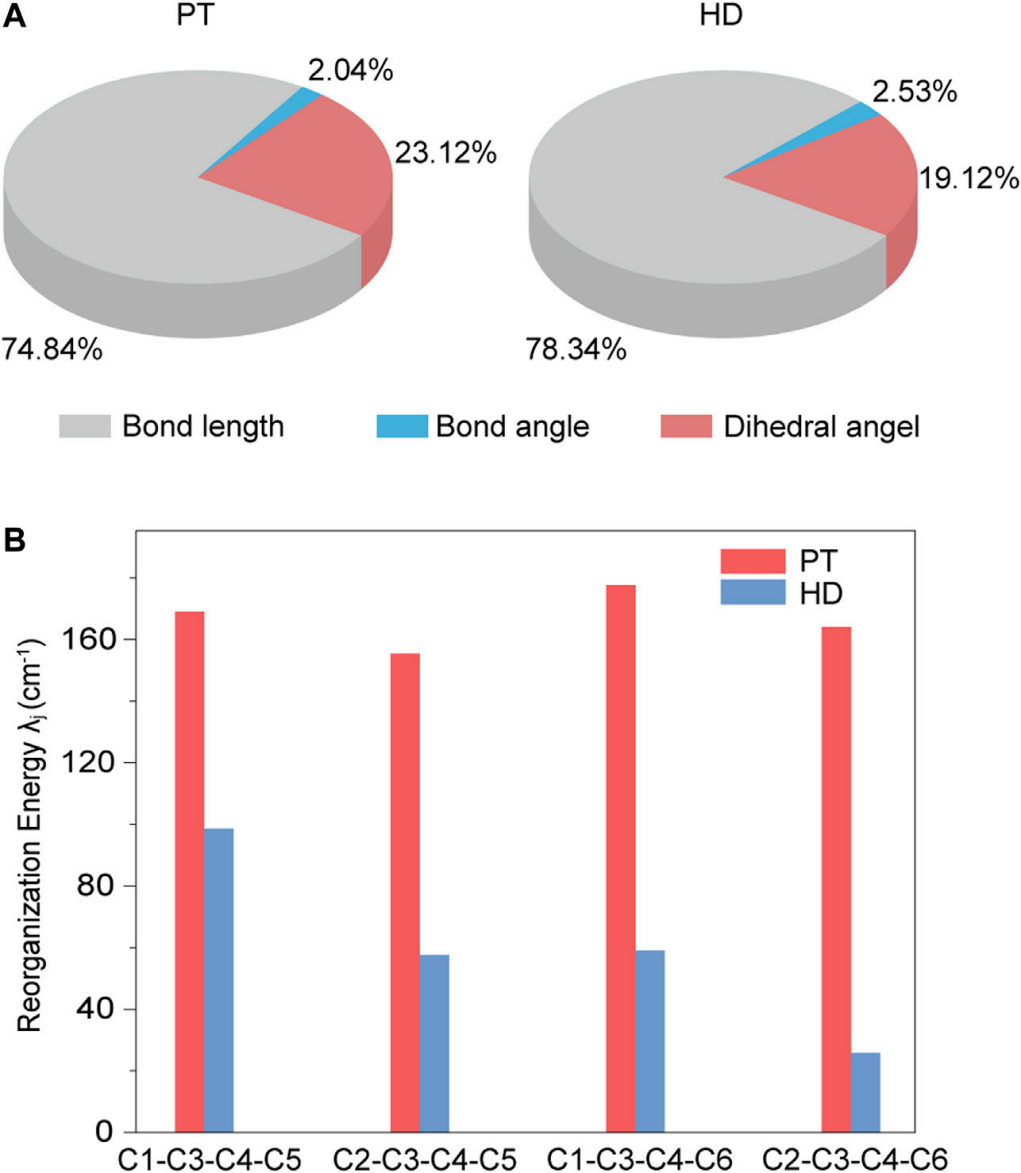
**(A)** Contributions to the total reorganization energy from the bond length, bond angle, and dihedral angle for PT and HD; **(B)** The mainly contributions to the reorganization energy from the different dihedral angles for PT and HD.

Next, by projecting the total *λ* into the internal coordinate relaxation of the compounds, Figure 4A showed that, going from PT to HD, the contributions from the bond length are increased from 74.84 to 78.34%, while the contributions derived from the bond angle have a tiny variation with a value of ca. 2.0%. Impressively, the contributions came from the dihedral angle associated with the torsional motions are reduced from 23.12% in PT to 19.12% in HD. Figure 4B further showed that such a decease is stemmed from the torsional vibrations between the central benzene and one nearby outer benzene (see Supplementary Table S4), for example, the reorganization energy from the dihedral angle of C1-C3-C4-C5 is reduced from 169.06 cm^−1^ to 98.61 cm^−1^, and for C2-C3-C4-C6, it is decreased from 164.03 cm^−1^ to 25.87 cm^−1^. Based on these features, we concluded that the nonplanar ethylene bridges can suppress the electron-vibration coupling of the torsional vibration modes, largely hindering the enlargement of nonradiative decay rate.

### Phosphorescence Spectra Under Ambient Condition

**FIGURE 5:**
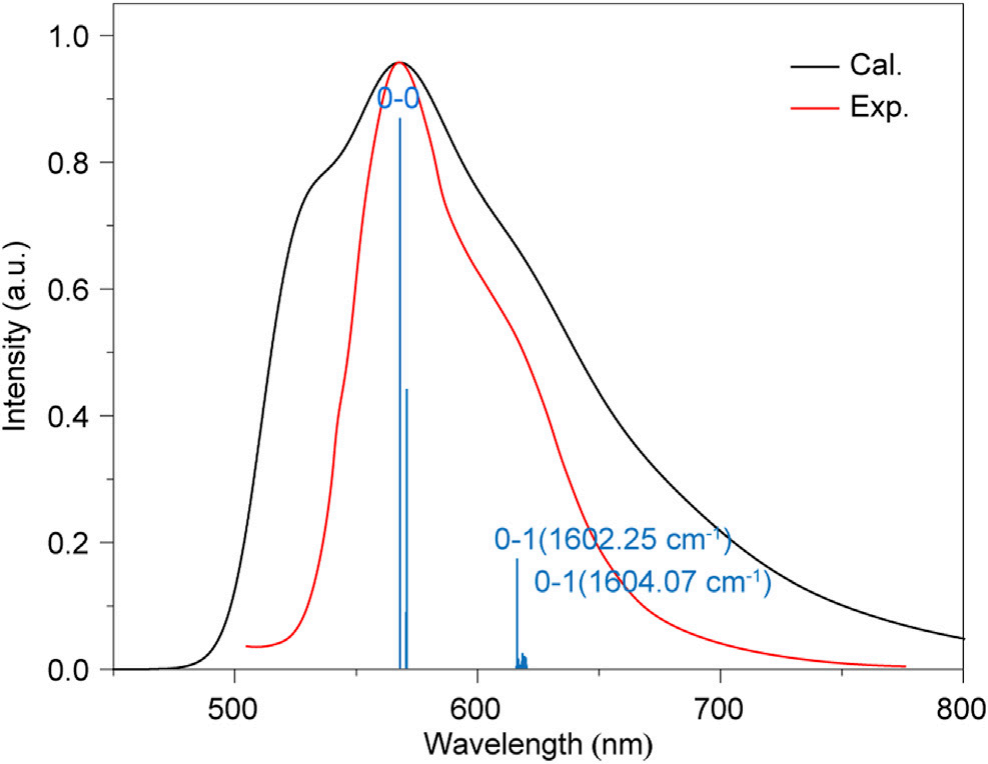
The calculated (black) and experimental (red) spectrum of phosphorescence for HD.

Beyond the RTP lifetime, we further calculated the phosphorescence spectra to verify our proposed ultralong RTP mechanism in nonplanar aromatic hydrocarbon. Figure 5 then displayed the simulated phosphorescence spectrum of HD to compare with the experiment. It is found that the theoretical results agree well with the experimental spectra; the peak maximum at around 570 nm corresponds to the 0–0 transition; and the shoulder peak at around 610 nm is mainly ascribed to the 0–1 transition for the C=C stretching with 1,602 and 1,604 cm^−1^. Namely, these results demonstrated our theoretical model is reliable, and it is enough to support the above proposed RTP mechanism.

## Conclusion

In summary, we explored the nature of low-lying excited states, excited-state decay rates and vibrationally resolved phosphorescence spectra by QM/MM models coupled with the TVCF rate formalism in the MOMAP program, to unravel the origin of the ultralong RTP in nonplanar aromatic hydrocarbon. Theoretical results demonstrated that, from PT to HD, the introduction of the ethylene bridges offer σ-electron to strengthen spin-orbit coupling (SOC) between singlet and triplet excited states, such a change not only largely accelerates the ISC process for efficient RTP, but also increases the radiative and nonradiative decay rates of T_1_→S_0_ process, hindering the ultralong phosphorescence lifetime of twisted HD molecule. Beyond the SOC effect, the ethylene bridges also reduce the electron-vibration coupling associated with the torsional vibration modes in low frequency regions (<300 cm^−1^), strongly reducing the nonradiative decay rate of T_1_→S_0_, and thereby facilitating to the ultralong RTP. Therefore, the synergistic effect of SOC and vibronic coupling caused by the ethylene bridges make a slight enlargement of the nonradiative decay rate for nonplanar HD, thereby generating an efficient RTP in twisted hydrocarbon with ultralong lifetime.

## Data Availability

The original contributions presented in the study are included in the article/Supplementary Material, further inquiries can be directed to the corresponding author.
