# Supplementary material for: Theoretical Insight Into the Ultralong Room-Temperature Phosphorescence of Nonplanar Aromatic Hydrocarbon
Source: Front Chem. 2021 Sep 6;9:740018. doi: 10.3389/fchem.2021.740018 (PMC8450344; doi:10.3389/fchem.2021.740018)
Supplement: Supplementary file 1 [file DataSheet1.docx]

Total words: 236; Total Figures: 3; Total Tables: 3

Supplementary Material

# Supplementary Figures and Tables

## Supplementary Figures


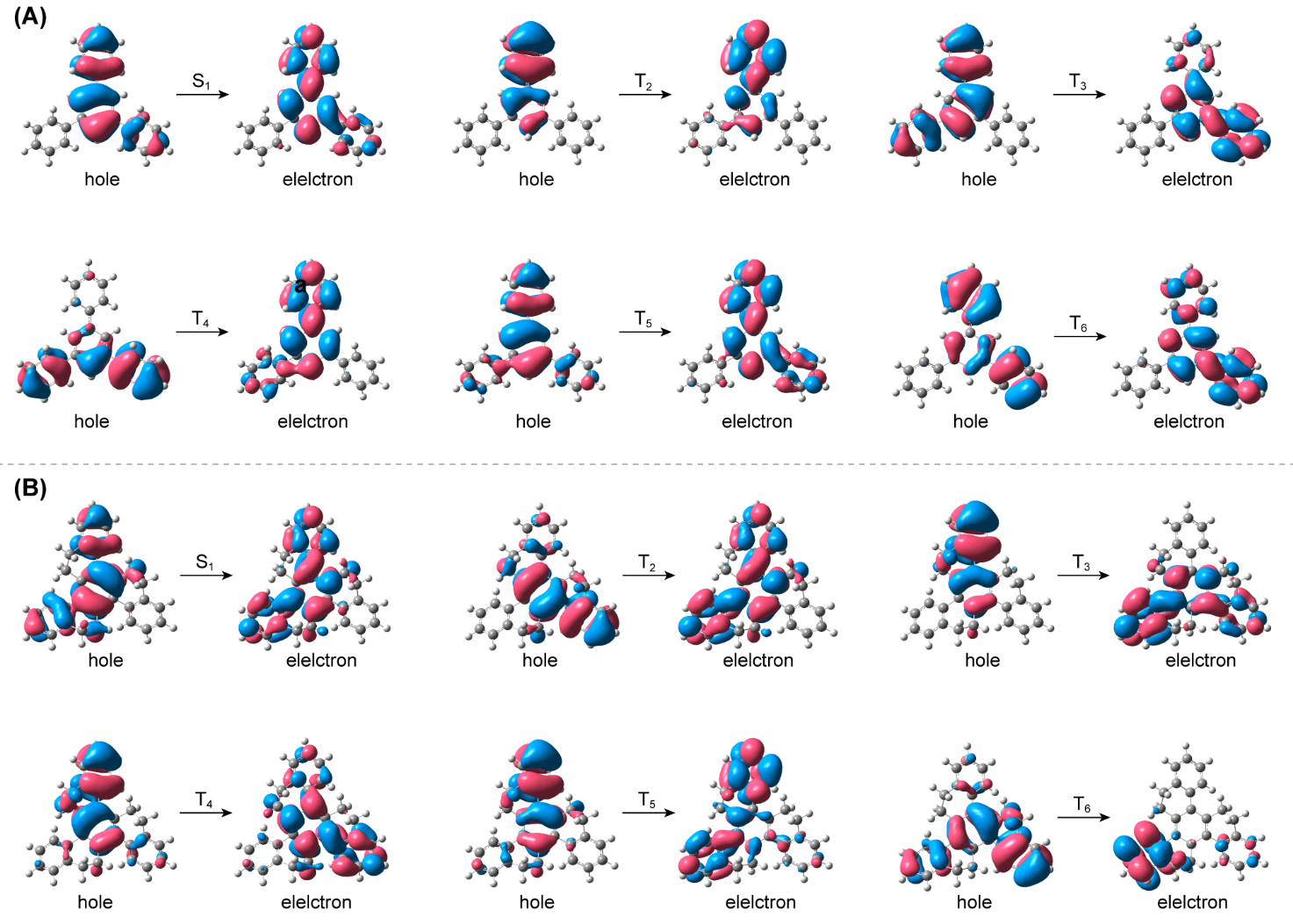


**Supplementary Figure S1.** The calculated NTOs of S_1_ and T_n_ (n=2-6) for (A) PT and (B) HD based T_1_-geometry.


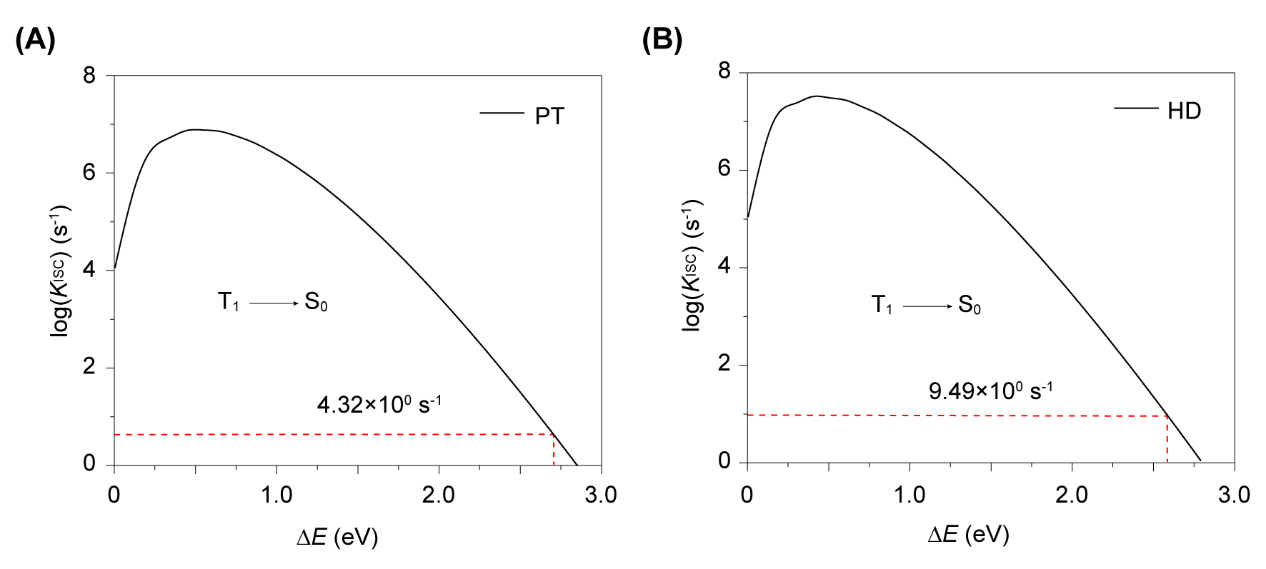


**Supplementary Figure S2.** Intersystem crossing rate constant spectra of T_1_ → S_0_ for (A) PT and (B) HD.


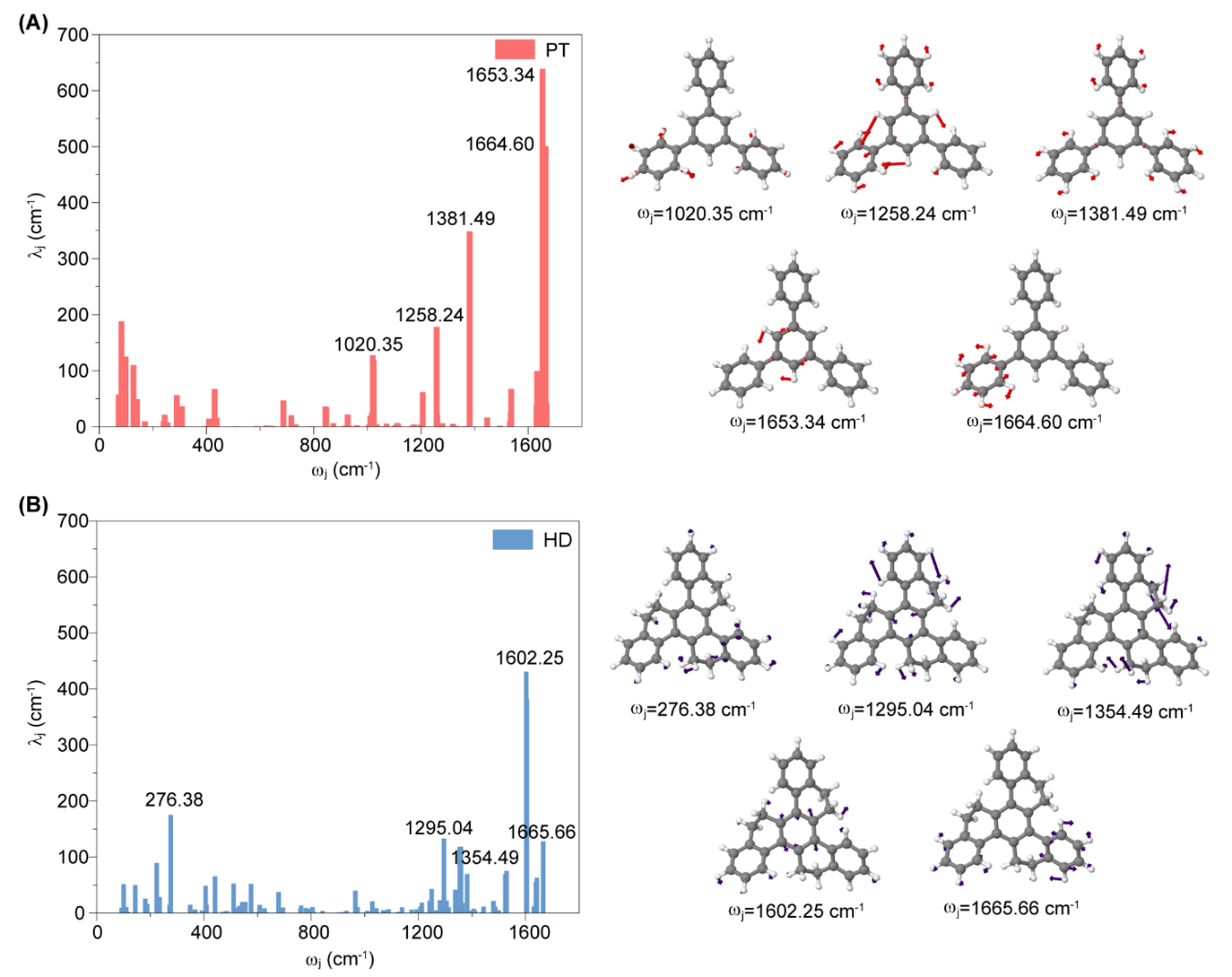


**Supplementary Figure S3.** Calculated normal-mode reorganization energy λ_j_ of T_1_ → S_0_ for (A) PT and (B) HD.

## Supplementary Tables

**Supplementary Table S1.** Calculated SOC matrix elements (ξ) of the low-lying excited states for PT and HD based on T_1_-geometry. The unit is cm^-1^.

| State | PT | | HD | |
| --- | --- | --- | --- | --- |
|  | ξ(S_0_, T_n_) | ξ(S_1_, T_n_) | ξ(S_0_, T_n_) | ξ(S_1_, T_n_) |
| T_1_ | 0.19 | 0.15 | 0.37 | 0.45 |
| T_2_ | - | 0.14 | - | 0.08 |
| T_3_ | - | 0.09 | - | 0.22 |
| T_4_ | - | 0.03 | - | 0.05 |
| T_5_ | - | 0.09 | - | 0.09 |
| T_6_ | - | 0.13 | - | 0.08 |

**Supplementary Table S2**. Calculated SOC matrix elements (ξ) of the low-lying excited states for PT and HD based on S_1_-geometry. The unit is cm^-1^.

| State | PT | | HD | |
| --- | --- | --- | --- | --- |
|  | ξ(S_0_, T_n_) | ξ(S_1_, T_n_) | ξ(S_0_, T_n_) | ξ(S_1_, T_n_) |
| T_1_ | 0.17 | 0.18 | 0.38 | 0.51 |
| T_2_ | - | 0.03 | - | 0.04 |
| T_3_ | - | 0.02 | - | 0.04 |
| T_4_ | - | 0.01 | - | 0.05 |
| T_5_ | - | 0.02 | - | 0.09 |
| T_6_ | - | 0.02 | - | 0.09 |

**Supplementary Table S3**. The proportion of (σ, π*) in the low-lying excited states for HD.

|  | S_1_ | T_1_ | T_2_ | T_3_ | T_4_ | T_5_ | T_6_ |
| --- | --- | --- | --- | --- | --- | --- | --- |
| (σ, π*) | 7.16% | 5.47% | 5.50% | 4.12% | 5.53% | 4.25% | 4.25% |

**Supplementary Table S4.** Calculated reorganization energy projected to the dihedral angles.

| Dihedral angles | λ_j_ (cm^-1^) | |
| --- | --- | --- |
|  | PT | HD |
| C1-C3-C4-C5 | 169.057 | 98.605 |
| C2-C3-C4-C5 | 155.349 | 57.654 |
| C1-C3-C4-C6 | 177.484 | 59.076 |
| C2-C3-C4-C6 | 164.031 | 25.867 |
